# Neural Correlates to Food-Related Behavior in Normal-Weight and Overweight/Obese Participants

**DOI:** 10.1371/journal.pone.0045403

**Published:** 2012-09-18

**Authors:** Alan Ho, James Kennedy, Anastasia Dimitropoulos

**Affiliations:** Department of Psychological Sciences, Case Western Reserve University, Cleveland, Ohio, United States of America; Duke University, United States of America

## Abstract

Two thirds of US adults are either obese or overweight and this rate is rising. Although the etiology of obesity is not yet fully understood, neuroimaging studies have demonstrated that the central nervous system has a principal role in regulating eating behavior. In this study, functional magnetic resonance imaging and survey data were evaluated for correlations between food-related problem behaviors and the neural regions underlying responses to visual food cues before and after eating in normal-weight individuals and overweight/obese individuals. In normal-weight individuals, activity in the left amygdala in response to high-calorie food vs. nonfood object cues was positively correlated with impaired satiety scores during fasting, suggesting that those with impaired satiety scores may have an abnormal anticipatory reward response. In overweight/obese individuals, activity in the dorsolateral prefrontal cortex (DLPFC) in response to low-calorie food cues was negatively correlated with impaired satiety during fasting, suggesting that individuals scoring lower in satiety impairment were more likely to activate the DLPFC inhibitory system. After eating, activity in both the putamen and the amygdala was positively correlated with impaired satiety scores among obese/overweight participants. While these individuals may volitionally suggest they are full, their functional response to food cues suggests food continues to be salient. These findings suggest brain regions involved in the evaluation of visual food cues may be mediated by satiety-related problems, dependent on calorie content, state of satiation, and body mass index.

## Introduction

In the U.S., data for 2003–2004 and 2005–2006 indicated that approximately two thirds of U.S. adults were either obese (body mass index (BMI) ≥30.0) or overweight (BMI of 25.0–29.9) [1]. A person being either overweight or obese causes pathological changes in the body and increases the risk for many chronic diseases such as heart disease, type 2 diabetes, and hypertension [2]. Although the basic biology of obesity development is not fully understood, it is recognized that obesity occurs primarily when energy intake exceeds energy expenditure. However, there are multiple etiologies for this imbalance, including biological and environmental, and as a result, the rising prevalence of obesity cannot be addressed by a single etiology.

As a result of the lack of understanding of the interplay among genetics, physiology, cognition, and behavior in the control of human body fat mass, preventative and therapeutic approaches to curb the obesity epidemic have had limited success [3], [4]. Currently, the primary form of treatment and management in obese individuals is behavioral therapy which combines instruction on dieting and physical activity in conjunction with behavioral strategies that facilitate replacing maladaptive behaviors with new eating and activity habits [5], [6]. Maladaptive behaviors associated with obesity include: lack of physical activity and food consumption patterns that have increased energy intake such as eating more than a standard sized meal, eating fast food, and never feeling satiated [7]. Physical activity helps attenuate the risk of mortality associated with excess adiposity [8], [9]. However, physical activity alone is of limited benefit because most individuals cannot find the time or motivation to expend enough energy to offset their usual energy intake [10]–[11]. Moreover, the craving for high-calorie food can be too much for one to follow a strict dietary plan [11]. Behavioral treatments alone have had limited success due to a person having overwhelming cravings for food. It has been argued that the difficulties in treating obesity stem from the intrinsic difficulty in overriding instinct and primal urges [12].

Neuroimaging studies to date have demonstrated that the central nervous system is important in regulating eating behavior even though region-specific roles are not yet fully understood [12]–[14]. Functional magnetic resonance imaging (fMRI) provides a noninvasive tool to investigate the etiology of eating behavior, the differences in activation of brain regions in different states of hunger, and possible targets for pharmacological treatments of obesity. The central nervous system plays a crucial role in adapting to changing energy demands. In addition, the brain plays a multitude of basic roles responsible for food intake regulation such as mediating hunger (defined as the physiological or metabolic state that results from a lack of energy or nutrients [15]), decisions involving food choice, the sensory and emotional pleasure associated with eating, and the control of energy metabolism [14]. To date, fMRI research of appetite and food motivation has shown differential activation patterns between individuals who are normal weight and those who are obese [12]–[14], [16]–[21]. For example, Martin et al. (2010) [22] found increased activations to food compared to nonfood cues in prefrontal and limbic regions, for obese compared to healthy weight individuals in both premeal and postmeal states. Groups may differ in their response to food cues after eating because of a greater risk of satiety dysfunction in those who are obese. Obese individuals may experience greater meso-limbic reward system activation in response to eating which increases the risk of overeating [23]. However, findings across studies show inconsistencies with respect to which regions are implicated in the role of eating and food motivation. Although this is possibly due to differences in methodology across studies, the exact mechanisms of food-related neural activation is not yet fully understood. While there are a number of brain regions responsible for ingestive behavior and appetite, research to date indicates that visual food cues activate food motivation and reward neural circuitry, including the hypothalamus, thalamus, striatum, insula, orbitofrontal cortex (OFC), dorsolateral prefrontal cortex (DLPFC), cingulate, amygdala, and hippocampus [12], [16], [17], [21], [24]–[27]. A recent study examining the neural correlates of food addiction symptoms in lean and obese women found participants with higher food addiction scores show greater activation in the striatum and dorsolateral prefrontal cortex in response to anticipated receipt of food and less activation in inhibitory regions in response to receipt of food, suggesting similar patterns of neural activation to those with substance dependence [28].

In the present study, we examined functional neural response to high- and low-calorie food visual cues in relation to food-related problems (preoccupation with food and difficulty with satiety) in normal weight and overweight/obese participants. Studies have been conducted on the relation of BMI to brain activity in response to food visual cues. However, to our knowledge, no studies to date have examined how variation in food related problems relate to neural functioning. While numerous factors such as cognitive factors, temporal factors (time of day), homeostatic regulation, hedonic eating, and food-related cues (i.e., taste, smell, texture, and appearance) influence motivation to eat, environmental visual cues are one of the initial and vital inputs that affect motivation to eat [29]. The decision to initiate food intake, how much to consume and when to stop eating is influenced by the interaction between homeostatic regulation and hedonic eating [30]. When consuming food is motivated by pleasure rather than homeostatic regulation, this behavior leads to poor appetite control and increased energy intake [31]. Ultimately, food related problems such as a preoccupation with food and an impairment of satiety influences an individuals’ decision to eat when both internal and external stimuli are processed. Food related problems may be a behavioral precursor to weight gain and greater insulin resistance. In a longitudinal study, young adults (age 18–30 years) who ate at fast-food restaurants more than twice a week compared to less than once a week had a two-fold increase in insulin resistance and gained an extra ten pounds 15 years later [32]. By assessing food related problems early in a young adults’ life regardless of BMI, it may be possible to prevent future health problems. Initially, we examined functional neural response across the entire sample and found no significant correlations with food-related problems. In light of this and given the substantial literature indicating differential response to food cues with respect to body mass index (BMI), our aim was to elucidate these correlates distinctly for these groups. Based on previous findings, we predicted food-related behavior will be positively correlated with neural activation in the striatum, OFC, DLPFC, insula, thalamus, hypothalamus, amygdala, and hippocampus in response to high-calorie food cues during hunger (premeal condition) in normal-weight and overweight/obese individuals. After eating, we expected correlations between eating behaviors and neural reward response to food cues in overweight/obese individuals but not among normal-weight participants as there is considerable evidence from our work and others of decreased response to food cues with meal ingestion for this group.

## Methods

### Ethics Statement

This research was approved by the University Hospitals Case Medical Center Institutional Review Board for Human Investigation. Written informed consent was obtained for all participants.

### Participants

Participants included 35 overweight/obese (OV/OB) and normal-weight (NW) individuals recruited from the Case Western Reserve University community. Using BMI classification, 21 participants were classified as overweight/obese (OV/OB) [mean(SD): 30.64 (4.1)] and 14 were normal-weight (NW) [mean(SD): 21.83 (1.4)]. All NW participants were right handed and all but 4 OV/OB participants were right-handed. Participants were in good health, had normal to corrected-normal vision, and were eligible for MRI scanning (i.e., free of ferromagnetic implants). Individuals who reported a history of psychiatric or neurological problems, significant weight loss or gain in the past 6 months, or head injury with loss of consciousness were not eligible to participate. All participants gave informed written consent and were financially compensated for their participation. This research was approved by the University Hospitals Case Medical Center Institutional Review Board for Human Investigation. Participants were recruited as part of a larger study examining hyperphagia and food related behaviors in individuals with Prader-Willi syndrome (PWS). Main group contrast findings have been recently reported [24]. Except for BMI, group characteristics did not differ significantly between groups (see Table 1).

**Table 1:** Participant Characteristics.

	NW(n = 14)	OV/OB(n = 21)	p-value
Age	24.5 (4.5)	24.4 (6.4)	0.949
BMI	21.83 (1.4)	30.64 (4.1)	0.0001
Gender (% female)	57.1	52.4	0.789
Race (% non-Caucasian)	28.6	33.3	0.774
Food Preference:			
High Cal (200+ calories)	3.85 (0.44)	3.85 (0.64)	0.996
Low Cal (<100 calories)	3.79 (0.41)	3.77 (0.47)	0.912
FRPQ:			
Preoccupation with food	7.5(2.41)	7.76(2.83)	0.778
Impairment of Satiety	15.07(1.59)	15.38(3.04)	0.697

For age, BMI, food preference, and FRPQ subscales, values are presented as mean (standard deviation). NW  =  normal-weight; OV/OB  =  overweight/obese. BMI indicates body mass index, based on height and weight obtained during testing.

### Eating Behavior

The Food-Related Problem Questionnaire (FRPQ) [33] is a survey originally designed to measure food-related problems in individuals with PWS. The 16-item survey includes 3 principal subscales: Preoccupation with food (FRPQ-FOOD), Impairment of satiety (FRPQ-SATIETY), and Composite negative behavior (FRPQ-CNB). The FRPQ-CNB subscale is composed of 3 minor subscales: takes and stores food, eats inedibles, and responds in an inappropriate way when food is not available or is restricted. The FRPQ-CNB subscale was not used in this study because the primary questions were not predicted to be relevant to the population of interest. FRPQ-FOOD (score range = 0–18) is made up of 3 questions: 1.) “How often do you compare the size or content of the meal with others”, 2.) “How often do you talk about food?”, and 3.) “Do you ever associate people and/or places with specific food items or occasions involving food?” FRPQ-SATIETY (score range = 0–30) is composed of 5 questions: 1. “After a normal size meal, how often do you still feel hungry?”, 2. “If you were tired, ill, or upset, how often would this result in you going without food?”, 3. “How frequently will you share food with others?”, 4. “How often do you feel full?”, and 5. “If given the opportunity, do you ever eat more than a standard sized meal?”. The FRPQ is scored on a 7-point Likert scale with responses ranging from 0 = ‘never’ to 6 = ‘always’. Reliability and validity has been performed with data from PWS and non-PWS samples [33]. The test-retest and inter-rater reliability are both .86. Subscale reliability ranges from .67–.85 and Cronbach’s Alpha for the total score is .87 in the combined PWS and non-PWS sample suggesting good internal consistency. Mean subscale scores on the validation sample were as follows: FRPQ-FOOD = 3.83(2.44) and FRPQ-SATIETY  = 13.25(5.88) [33]. Although the FRPQ was developed for people with PWS, the FRPQ can be used with non-PWS populations as the questions are not specific to PWS behavior and can inform eating behavior in the general population.

### Procedure

Participants were scanned between 12 and 2pm consecutively for a premeal and a postmeal scan. As part of the larger study, scanning was constrained by the study parameters regarding participants with PWS. Thus, scanning on separate days (and as a result, counterbalancing premeal and postmeal state) was not feasible. Participants were asked to eat a light breakfast before 8∶00am prior to their appointment on the day of their scans and to refrain from eating until the experimental procedure was completed. Prior to scanning, participants underwent neuropsychological testing (as part of the larger study not reported here) and training on the functional tasks. Height, weight, and a food preference assessment were also obtained during this time. The food preference assessment was administered to obtain a measure of high- and low-calorie food preference for each participant. Participants were shown colored flash cards of 74 foods and were required to rate each food on a 5-point Likert scale from ‘dislike extremely’ to ‘like extremely.’ Foods included desserts, meats, fruits, vegetables, junk food, breads, and pastas. The photographs for the food preference assessment were different from the images used in the fMRI task. High-calorie and low-calorie food preference ratings did not differ within or between groups (see Table 1).

Following the premeal scan, participants were given a meal prepared by the Dahms Clinical Research Unit at University Hospitals standardized to provide approximately 750 calories and consisting of a sandwich (choice of turkey, roast beef, or vegetarian), carton of milk, a serving of fruit, and either a side of a vegetable or cottage cheese. The postmeal scan typically began within 30 minutes of meal termination. Immediately before and after premeal and postmeal scans, participants answered the question, ‘How hungry are you right now?’ on a scale ranging from 0–8 with 0 being ‘not hungry at all’ to 8–‘extremely hungry’. It should be noted that while participants were instructed to eat until satiated, a direct measure of satiation was not administered but was indirectly inferred by change in hunger status.

### fMRI Task

fMRI blood oxygen level dependent (BOLD) responses were measured in a block design experiment with two runs performed during fasting (premeal) and two runs performed after eating a standardized meal of 750 calories (postmeal). During each functional run, participants performed a same/different perceptual discrimination task, in which two color photographic images were presented side-by-side and the participant would indicate by a button press whether the objects were the same or different. The same/different task parameters were selected to ensure participants were attending to the stimuli and to drive the perceptual systems engagement during food and nonfood discrimination during a hunger state. Images were presented in counterbalanced blocks corresponding with the 3 image types: high-calorie foods, low-calorie foods, and furniture. Low-calorie images averaged 50 cal (per serving size) and included pictures such as fresh vegetables and fruits. High-calorie images averaged 200 cal (per serving size) and included pictures such as chocolate chip cookies, French fries, and pizza. Each image consisted of only one type of food or object and were presented only once during the scan. Task design consists of eight blocks (21 seconds each, with a 14-second rest between blocks) with each block consisting of six image pairs presented for 2250 milliseconds (inter-stimulus interval: 1250 ms).

### fMRI Data Acquisition

MRI scanning was performed on a 4.0T Bruker MedSpec MR scanner using an 8-channel phase array transmit receive head coil located in the Case Center for Imaging Research. Participants’ heads were immobilized by placement of foam padding around the head. Functional images were acquired using a gradient-echo single-shot echo-planar sequence over 35 axial sequence slices aligned parallel to AC-PC plane with an inplane resolution of 3.4×3.4×3 mm (TR = 1950, TE = 22 ms, flip angle = 90). Bold activation data was acquired during two runs (5∶01 minutes each, 157 echo-planar images per run) each MRI session. Images were back-projected onto a translucent screen placed near the end of the MRI scanner and were viewed through a periscopic prism system on the head coil. 2D T1-weighted structural images (TR = 300, TE = 2.47 ms, FOV = 256, matrix = 256×256, flip angle = 60 degrees, NEX = 2), 3 mm thick, positioned in the same plane and slice locations as the echo-planar data for in-plane registration and a high resolution 3D structural volume (3D MPRAGE, contiguous, sagittal acquisition, 176 slice select partitions, each with 1 mm isotropic voxels, TR = 2500, TE = 3.52 ms, TI = 1100, FOV = 256, matrix = 256×256, flip angle = 12 degrees, NEX = 1) were collected during the initial (premeal) session.

### fMRI Data Analysis

Image processing, analyses, and tests of statistical significance were performed using Brainvoyager QX [34]. Preprocessing steps included trilinear three dimensional motion correction, spatial smoothing using a Gaussian filter with a full-width half-maximum value of 7 mm, and linear trend removal. Motion correction parameters were added to the design matrix and motion >2 mm along any axis (x, y, or z) resulted in the discard of that data (<1% discarded for this sample). Data for each individual was aligned with high-resolution 2D and 3D anatomical images for display and localization. The individual data sets underwent piecewise linear transformation into a proportional 3D grid defined by Talairach and Tournoux [35] and were coregistered with the high-resolution 3D data set and resampled to 3 mm^3^ voxels.

The normalized data sets were then entered into a second level analysis in which functional activation was examined using a random effects general linear model (GLM) analysis for the premeal scans and for the postmeal scans. GLMs contrasting the three experimental conditions: high-calorie foods, low-calorie foods, and object (furniture) were examined for the OV/OB group and NW group separately. For both participant groups, the statistical threshold for regions previously identified in the literature as part of the food motivation and reward neural circuitry (OFC, DLPFC, insula, cingulate, hypothalamus, thalamus, striatum, amygdala and hippocampus) was set at p<.005 with a minimum cluster extent of >5 contiguous voxels. For whole brain examination, the statistical threshold was set at p<.001, cluster threshold >5 with false discovery rate (FDR) correction at p<.05. The cluster threshold correction technique controls false positives with a relative sparing of statistical power [36]. Statistical thresholding in fMRI research has become increasingly conservative over the past decade where there has become an unintended negative bias towards studying only large effects (sensory and motor processes) rather than small effects (cognitive and affective processes). Thus, a combined intensity and non-corrected cluster size threshold was used to achieve a desirable balance between Type I and II error rates [37].

Mean signal intensities from regions that met statistical threshold for significance were then extracted giving information on the magnitude of activation of the BOLD signal (beta values). This data was then entered into SPSS 17.0 (Chicago, IL, USA) for additional analysis. Upon extraction, beta contrasts were computed for each calorie condition vs. nonfood objects during each hunger state (high-calorie – object, premeal state; low-calorie – object, premeal state; high-calorie – object, postmeal state; low-calorie – object, postmeal state). Pearson correlations were conducted to examine the relation between activation from significant regions and the two FRPQ subscale scores (FRPQ-SATIETY and FRPQ-FOOD). Correlations were considered significant if they exceeded a threshold of p<0.03 for food-related regions described above and a threshold of p<0.005 for other regions.

## Results

### Behavioral Data

Hunger ratings prior to each scan session differed significantly between premeal and postmeal conditions for both NW [premeal scan mean(SD) = 4.32(1.38); postmeal scan = .43(.85); t = 11.7, p<.000] and OV/OB participants [premeal scan mean(SD) = 4.71(1.58); postmeal scan = .43(.87); t = 13.64, p<.000]. There were no significant between-group differences in hunger ratings during premeal (p = .495) or postmeal (p = .834). These findings indicate the meal manipulation was effective, with both groups reporting decreased hunger from premeal to postmeal sessions. Thus, for the purposes of this study, the premeal scan was considered the hunger condition and the postmeal scan was interpreted as satiated given the findings from the hunger ratings. Task accuracy during the functional runs (same/different object task) was greater than 90% for all task conditions and did not differ significantly by scan session or group [NW: premeal mean% = 97.9(1.7); postmeal = 98.7 (1.4); OV/OB: premeal  = 97.5(2.3); postmeal  = 99.0(1.6)]. FRPQ subscale scores (FRPQ-SATIETY and FRPQ-FOOD) did not differ between groups (Table 1). Correlational analyses were performed between BMI and FRPQ subscale scores across weight groups and separately for NW and OV/OB. No significant correlations were found between BMI and either FRPQ subscale for the full sample or the OV/OB group. For the NW group, BMI was positively correlated with FRPQ-SATIETY (r = .738, p<.01).

### Correlates with Functional Activation

**Figure 1:**
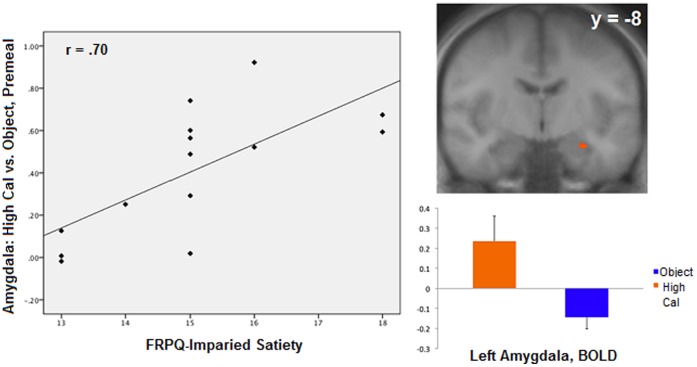
Normal-weight group (n = 14). *Left*: plot of correlation between high-calorie vs. nonfood objects contrast during premeal scan in the left amygdala and FRPQ Impairment of Satiety subscale score. *Right* top: activation of left amygdala to high-calorie food vs. nonfood objects (Talairach coordinates x, y, z: −26, −8, −14, t = 4.31, p<.0008, cluster threshold >5 voxels, FDR corrected at p<.05). Right and left are reversed by radiologic convention. *Right bottom:* magnitude of average activation for high-calorie foods and nonfood objects for right amygdala at premeal. Beta values reflect BOLD contrast averaged across voxels in region for condition type.

**Figure 2:**
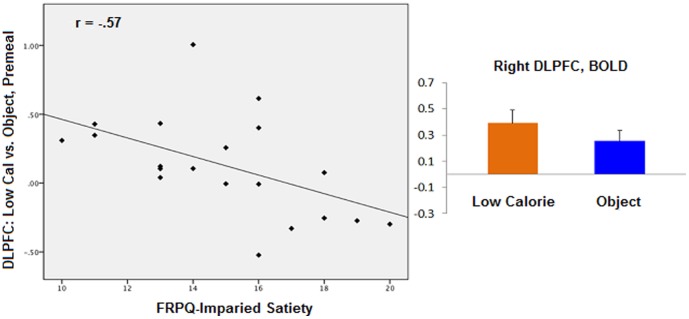
Overweight/obese group (n = 21). *Left*: plot of correlation between low-calorie vs. nonfood objects contrast during premeal scan in the right DLPFC and FRPQ Impairment of Satiety subscale score. *Right*: magnitude of average activation for low-calorie foods and nonfood objects in the right DLPFC (Talairach coordinates x, y, z: 49, 14, 28) at premeal scan. Beta values reflect BOLD contrast averaged across voxels in region for condition type.

**Figure 3:**
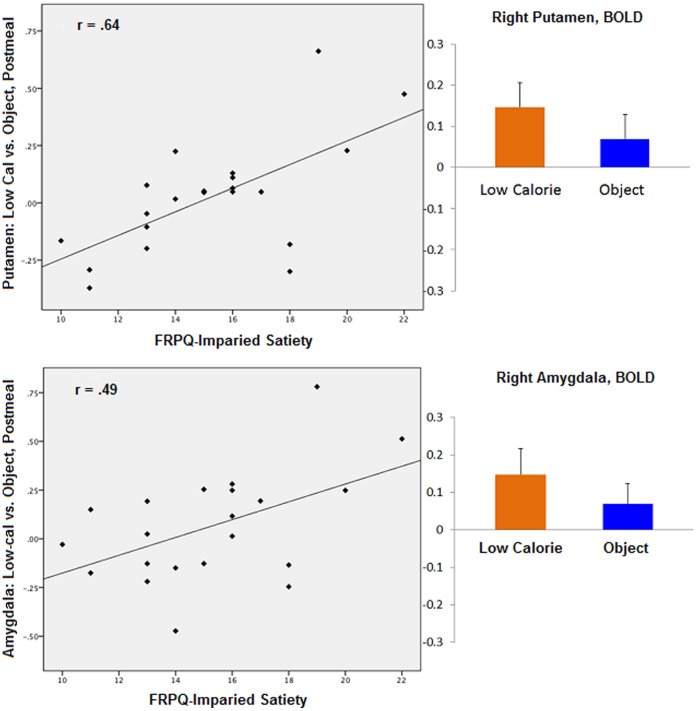
Overweight/obese group (n = 21). *Top Section*, *left*: plot of correlation between low-calorie vs. nonfood objects contrast during postmeal scan in the right putamen and FRPQ Impairment of Satiety subscale score. *Top Section, right*: magnitude of average activation for low-calorie foods and nonfood objects in the right putamen (Talairach coordinates x, y, z: 27, 3, −4) at postmeal scan. Beta values reflect BOLD contrast averaged across voxels in region for condition type. *Bottom Section*, *left*: plot of correlation between low-calorie vs. nonfood objects contrast during postmeal scan in the right amygdala and FRPQ Impairment of Satiety subscale score. *Bottom Section, right*: magnitude of average activation for low-calorie foods and nonfood objects in the right amygdala (Talairach coordinates x, y, z: 24, −7, −9) at postmeal scan. Beta values reflect BOLD contrast averaged across voxels in region for condition type.

During the premeal scan, activity in the left amygdala (TAL coordinates: x = −26, y = −8, z = −14; r = .70, p = .005) was positively correlated with FRPQ-SATIETY during the high-calorie vs. object contrast for the normal-weight group (Figure 1). Left amygdala activation during the same contrast was not significantly related to FRPQ-SATIETY in the OV/OB group (TAL: −18, −2, −11, r = .15, p = .50). No significant correlations between brain and FRPQ subscale scores were found with any contrast in the postmeal state. For the OV/OB group, premeal activity in the right DLPFC (TAL: 49, 14, 28; BA 9; r = −.56, p = .007) was negatively correlated with FRPQ-SATIETY in the low-calorie vs. object contrast (Figure 2). DLPFC activation during the same contrast in the NW group was not significantly related to FRPQ-SATIETY (TAL: 49, 14, 28, r = .30, p = .29). After eating, activation in the putamen (TAL: 27, 3, −4; r = .64, p = .002) and the amygdala (TAL: 24, −7, −9); r = .49, p = .02) were positively correlated with FRPQ-SATIETY in the low vs. object condition (Figure 3). Amygdala activation during the same contrast was not significantly related to FRPQ-SATIETY in the NW group (TAL: 24, −7, −9, r = .25, p = .37). There was no significant activation in the putamen region for the low vs. object condition for the NW group with which to extract for correlation analysis with the FRPQ measures.

## Discussion

In the present study, neural activation in response to food cues was found to correlate with self-report of impaired satiety depending on calorie content, state of satiation, and obesity status. Before eating, activation of the amygdala was positively correlated with self-report of impaired satiety in response to high-calorie vs. object cues among normal-weight participants. In addition, as hypothesized, no significant correlations between neural activation and behavior report were found postmeal in normal-weight individuals. In overweight/obese participants, significant correlations between brain activation and self-report of behavior were found in the dorsolateral prefrontal cortex (DLPFC) before eating and the putamen and amygdala at postmeal. Significant correlations between eating behavior and food-related and reward neural circuitry suggest that these brain areas may play a significant role in mediating food motivation and overeating.

### Normal-weight Group

During fasting, activation of the left amygdala was positively correlated with impaired satiety scores. Those with more satiety-related problems had greater activity in the amygdala in response to high-calorie food in contrast to objects. In monkeys, lesion to the amygdala causes indiscriminate sampling of nonfood and food items alike [38] and altered food preferences [39]. The amygdala also encodes anticipatory food reward; the anticipation of receipt of a palatable food as opposed to unpalatable food results in greater activation in the amygdala [40]. Other research indicates anticipation of a pleasant drink resulted in greater activation in the amygdala during fasting but not satiation [41]. In addition, in animal research, the greatest dopaminergic activation occurs as rats anticipate the produced food reward and this activation decreases once the reward is obtained [42]. Thus, our finding may suggest that individuals who tend to eat more than a normal size meal or feel hungry more often, may give desirable, high-calorie foods greater reinforcing value and emotional salience when hungry. The amygdala has extensive visceral and sensory anatomic connections [43] and thus, alterations by internal states such as hunger may modulate one’s motivation. Response of the amygdala to high-calorie vs. object cues may vary according to the current motivational state of the individual to the sight and thought of food with those scoring higher in impaired satiety possibly having a higher motivational state while fasting due to placing a higher reward value to anticipated food intake. This finding indicates that the amygdala may play a significant role in problematic eating behavior when feeling hungry. When a desirable high-calorie food is presented, individuals with higher impaired satiety scores may allow retrieval of past pleasurable food experiences and give greater emotional salience to highly desirable food. In those with higher impaired satiety scores, amygdala activity is amplified when one is hungry. However, while there seem to be differences in subjective reward value associated with impaired satiety scores, this amplification seems to be tempered after eating a 750 kcal meal among normal-weight individuals. Thus, a normal sized meal may placate satiety problems a lean individual may have, unlike overweight/obese individuals. While not measured in this study, it is possible these individuals with higher impaired satiety scores are more prone to eating high calorie foods until satiated. However, while a young adult with satiety dysfunction may remain lean, their BMI and health (i.e., insulin resistance) may be at increased risk as they age.

### Overweight/obese Group

During fasting, activity in the DLPFC in response to low-calorie vs. object food cues was negatively correlated with impaired satiety scores. The DLPFC is implicated in high-level executive functions and decision-related processes, cognitive control, and regulating one’s emotion [44], [45]. Moreover, activation of the DLPFC is related to perceived reward value as Hare and colleagues [46] found that the ‘goal value’ of food correlated with activation of this brain region. In congruence with findings from the results of this study, the DLPFC may be part of an inhibitory system that controls ones’ feeling of satiety through emotion regulation in addition to mediating the reward value of food. This may explain why individuals scoring lower in satiety impairment preferentially activate this inhibitory system. We were somewhat surprised to find this correlation to the low-calorie images vs. object contrast and not the high-calorie vs. object. One explanation may be that overweight/obese individuals have a different mental representation of what food is appropriate for them to eat in comparison to normal-weight individuals. Food preference findings indicated that there were no significant differences between groups in what they find palatable. However, there may be a distinction between ‘liking’ (palatability) and ‘wanting’ (appetitive or incentive motivation). The decision to eat or refrain from eating can be based on learning to anticipate the consequences of food intake [47]. For example, just because one ‘likes’ cheesecake because he knows it tastes good does not necessarily mean he may wish to eat it due to a decrease in incentive motivation (e.g., possible diet restriction or worrying about the possible negative health consequences of eating a high-calorie dessert). Thus, this preferential activation of the DLPFC to low-calorie images in overweight/obese individuals may be a reflection of their incentive motivation, ‘wanting’, to low-calorie foods when hungry, not what they find most palatable. Similarly, restrained eaters make a conscious effort to restrict food intake to control their body weight [48].

After eating, activity in both the putamen and the amygdala were positively correlated with impaired satiety scores in response to low-calorie food cues contrasted with nonfood objects. The amygdala encodes anticipatory food reward [40], [41] and damage to this region can result in hyperphagia [49], [50]. Functional activation of the amygdala has been previously shown to vary with weight, satiation, and calorie content [51]–[54]. The striatum has long been linked to reward processing. Volkow et al. [55] found significant increases in dopamine levels in the dorsal striatum in hungry individuals when exposed to food stimuli suggesting that the putamen is associated with motivation to eat. In addition, the amount of dopamine released in the dorsal striatum is correlated with food pleasantness [56]. Our findings suggest that even though the hunger rating scale given to participants before and after their postmeal scan indicate these individuals report very little hunger after eating, motivation to eat may still be present. Those who report to be less likely to be satiated after a normal sized meal on the behavioral measure had greater activity in these limbic regions postmeal. This finding suggests that there may be a disconnect between parts of the brain involved in decision-making and the orexigenic areas of the brain. The conflict between controlling food intake and the hedonic response to food is a battle frequently fought by overweight/obese individuals.

### Limitations and Conclusions

The current findings indicate further investigation into food-related problem behavior is warranted, regardless of weight classification. While the current sample size is adequate for a neuroimaging study, for correlation analysis, a larger sample size is preferable and thus these findings should be interpreted as preliminary. In addition, the FRPQ has greater relevance in research and clinical practice to appraise the food-related problems in individuals with PWS and thus not every subscale was appropriate for use with a nonclinical sample. Future examinations should replicate these findings with eating behavior measures designed for use with the general population. That said, it should be noted that there were very few regions found to correlate with the FRPQ scores, and of those that reached significance, all were in regions previously implicated in food-related processing. In addition, BMI correlated highly with FRPQ-Satiety among NW participants but not with the OV/OB group. While this was somewhat surprising, it may indicate which NW individuals may be at greater risk of becoming overweight or health problems related to poor eating habits. Like most other obesity fMRI studies, we did not identify the cause of obesity for each participant, yet self-report of eating problems did not vary by BMI. This may indicate that once overweight, variability in eating habits across individuals who are OV/OB may decrease. It should also be noted that beta contrasts were computed for each contrast condition (e.g., high-calorie vs. nonfood, postmeal, low-calorie vs. nonfood, premeal, etc.) if a region met statistical threshold criteria in response to one specific contrast. Each of these were then examined for correlations with the FRPQ variables. Thus, the magnitude of difference between functional conditions varied by contrast and some regions were not viewable on the group GLM map. As such, these findings should be considered preliminary. Despite this, we feel these findings warrant dissemination to highlight distinctions between food-related processing and eating behavior in normal and OV/OB samples. Lastly, given the nature of the passive viewing paradigm, future research may seek to incorporate olfactory or gustatory senses, which may provide a more visceral sensory experience.

Despite these limitations, findings presented here suggest that differences in food related behavior may mediate activation in areas of the brain important for food motivation, subjective reward, and emotional regulation. In addition, food-related behavior is not only relevant for individuals who are overweight or obese. In normal-weight individuals, increased activity to high-calorie food cues in the amygdala during hunger suggests behaviors related to impaired satiety may reflect dysfunction in how the amygdala encodes anticipatory food reward, particularly with respect to highly palatable foods. However, this amplification is tempered postmeal. These findings suggest that the individuals with normal weight who are more likely to report poor eating habits may be at risk for future eating-related health problems given their greater neural response to food cues than those who report fewer problems. In overweight/obese individuals, the DLPFC may play an important role in whether one ‘thinks’ about food more than others and whether one ‘feels’ full or not after eating a 750kcal meal. Moreover, among overweight/obese individuals, reward response after eating may be mediated by problematic food habits. As this sample consists of individuals who are overweight or obese for a variety of reasons, it is reasonable to suggest that those with poorer food habits are differential motivated by food than those with better eating behavior. Instead of curbing food intake due to internal hunger cues, overweight and obese individuals may regulate food intake due to psychological cues.

In summary, this study has shown that variability in satiety-related problems has unique correlations with activation in specific brain regions known to be involved in food related behavior both in normal-weight and overweight/obese individuals. We show preliminary evidence that even within a normal weight sample, eating habits mediate activation to food cues. This variance among those with normal-weight may indicate characteristics that may put individuals at risk for eating-related health problems and/or obesity in the future. However, as these findings are preliminary and correlations between food-related behavior and response to food cues were not found after eating in the normal weight group, further examination is warranted. As expected, food-related behavior was salient for individuals who are overweight/obese before and, more importantly, after eating. These findings extend previous work in obesity research indicating differential neural response to food cues between BMI groups may be influenced by individual eating habits. Improved understanding of the correlation between food-related behavior and neural activation to appetitive stimuli may lead to a greater understanding of food motivation, reward, and mechanisms of overeating.
